# TNF-*α* Enhances the Therapeutic Effects of MenSC-Derived Small Extracellular Vesicles on Inflammatory Bowel Disease through Macrophage Polarization by miR-24-3p

**DOI:** 10.1155/2023/2988907

**Published:** 2023-02-28

**Authors:** Huikang Xu, Jiamin Fu, Lijun Chen, Sining Zhou, Yangxin Fang, Qi Zhang, Xin Chen, Li Yuan, Yifei Li, Zhenyu Xu, Charlie Xiang

**Affiliations:** ^1^State Key Laboratory for Diagnosis and Treatment of Infectious Diseases, National Clinical Research Center for Infectious Diseases, Collaborative Innovation Center for Diagnosis and Treatment of Infectious Diseases, The First Affiliated Hospital, Zhejiang University School of Medicine, Hangzhou, Zhejiang 310003, China; ^2^Research Units of Infectious Disease and Microecology, Chinese Academy of Medical Sciences, Hangzhou, Zhejiang 310003, China; ^3^Innovative Precision Medicine (IPM) Group, Hangzhou, Zhejiang 311215, China

## Abstract

Human menstrual blood-derived mesenchymal stem cells (MenSCs) and their secreted small extracellular vesicles (EVs) had been proven to relieve inflammation, tissue damage, and fibrosis in various organs. The microenvironment induced by inflammatory cytokines can promote mesenchymal stem cells (MSCs) to secrete more substances (including EVs) that could regulate inflammation. Inflammatory bowel disease (IBD) is a chronic idiopathic intestinal inflammation, the etiology and mechanism of which are unclear. At present, the existing therapeutic methods are ineffective for many patients and have obvious side effects. Hence, we explored the role of tumor necrosis factor *α*- (TNF-*α*-) pretreated MenSC-derived small EV (MenSCs-sEV^TNF-*α*^) in a mouse model of dextran sulfate sodium- (DSS-) induced colitis, expecting to find better therapeutic alterations. In this research, the small EVs of MenSCs were obtained by ultracentrifugation. MicroRNAs of small EVs derived from MenSCs before and after TNF-*α* treatment were sequenced, and the differential microRNAs were analyzed by bioinformatics. The small EVs secreted by TNF-*α*-stimulating MenSCs were more effective in colonic mice than those secreted directly by MenSCs, as evidenced by the results of histopathology analysis of colonic tissue, immunohistochemistry for tight junction proteins, and enzyme-linked immunosorbent assay (ELISA) for cytokine expression profiles in vivo. The process of MenSCs-sEV^TNF-*α*^ relieving colonic inflammation was accompanied by the polarization of M2 macrophages in the colon and miR-24-3p upregulation in small EVs. In vitro, both MenSC-derived sEV (MenSCs-sEV) and MenSCs-sEV^TNF-*α*^ reduced the expression of proinflammatory cytokines, and MenSCs-sEV^TNF-*α*^ can increase the portion of M2 macrophages. In conclusion, after TNF-*α* stimulation, the expression of miR-24-3p in small EVs derived from MenSCs was upregulated. MiR-24-3p was proved to target and downregulate interferon regulatory factor 1 (IRF1) expression in the murine colon and then promoted the polarization of M2 macrophages. The polarization of M2 macrophages in colonic tissues then reduced the damage caused by hyperinflammation.

## 1. Introduction

Inflammatory bowel disease (IBD) is a nonspecific and refractory chronic intestinal disease, which is also known as green cancer because of its prolonged course and serious decline in patients' quality of life [1–3]. Abdominal pain, diarrhea, hematochezia, and weight loss are the primary clinical symptoms of patients with this disease. The incidence of IBD had increased dramatically in some developing countries in Asia [4]. The number of IBD patients in China was also increasing year by year, and it had been reported that it could reach 150,000 in 2025 [5]. A retrospective study of IBD published in the *Lancet* in 2017 showed that the incidence in the United States and Europe had reached a stable level, while non-Western countries were experiencing a significant increase in new cases [6]. The direct mechanism of IBD pathogenesis was unclear, but environments, genetics, and intestinal microbiota had been reported to be involved [7, 8]. The uncertain pathology and mechanisms of the disease led to limited research into the remedy of IBD. Traditional therapies, including immunosuppressive agents and biological agents, were prone to develop therapeutic drug resistance, with less significant efficacy and obvious side effects [9]. Anti-tumor necrosis factor (TNF) agents are the most advanced treatment method nowadays, but it is ineffective for up to 40% of patients [10]. Therefore, there is an urgent need to find out a more appropriate alternative therapy.

Mesenchymal stem cell (MSC) injection is a new treatment for IBD, and some researchers had carried out clinical trials of MSC administration for Crohn's disease [11]. The therapeutic effect of MSCs for inflammatory diseases relies on two main aspects: one is that MSCs enter the body and home to the damaged site and differentiate into damaged cells [12]; the other is that MSCs regulate inflammation by secreting extracellular vesicles (EVs) [13]. However, it is difficult for MSCs to survive in tissues for a long time after entering the human body, and their ability to differentiate into colonic tissues is minimal [14]. Thus, we hypothesized that MSCs relieved colitis mainly by secreting certain anti-inflammatory or prorepair substances in EVs.

MSCs and their derived EVs played a functional role in the treatment of inflammatory diseases in previous studies [15–21]. The umbilical cord, bone marrow, and adipose-derived MSC-exosomes had been shown to have efficacy on IBD, with different therapeutic mechanisms [22–24]. Exosomes derived from MSCs can play a similar role in immunomodulation and injury repair as MSCs. Exosomes are small vesicles between 30 and 200 nm in diameter, rich in protein and complex ribonucleic acid (RNA) [25]. Minimal information for studies of extracellular vesicles 2018 (MISEV2018) defined EVs as all particles naturally secreted by cells, including exosomes released by late endosomes. They have a bilayer structure and are not self-replicating, and small EVs are defined as vesicles that are less than 200 nm in size [26]. The exosomes mentioned in previous studies in 2018 mostly were small EVs. EVs have the advantages of small size, direct action, and easy storage, and they do not face safety problems such as pulmonary embolism caused by cell therapy [27, 28]. In addition, many studies had demonstrated that EVs from MSCs exchange information with other cells through the small RNAs in them and further alter gene expression [29–32]. The small RNAs in these studies were mainly microRNAs, which are noncoding single-stranded RNA molecules with a length of about 22 nucleotides encoded by endogenous genes [33, 34]. Human menstrual blood-derived mesenchymal stem cells (MenSCs) are derived from the female endometrium and excreted from the female body through menstrual blood. They are characterized by strong self-replication and directional differentiation and low immunogenicity. Because of its low immunogenicity, it rarely arose immune rejection when it is used as allogeneic cell therapy. The advantages of MenSCs include easy access, high cell proliferation rates, and no ethical issues [35–37]. In previous studies, MenSCs and their small EVs can alleviate diseases such as tumors, pulmonary fibrosis, and liver failure [37–40]. Researchers had shown that MSCs regulate inflammation bidirectionally and inhibit inflammation only in the context of inflammation [41, 42]. Trace amounts of proinflammatory cytokines, such as interferon *γ* (IFN-*γ*) and TNF-*α*, contributed to the activation of anti-inflammatory effects of MSCs [43, 44].

When the colon tissue is damaged, macrophages play a significant role in destroying foreign microorganisms, removing necrotic tissues and debris, and tissue remodeling and regeneration [2]. Macrophages in the inflammatory environment include the “classical-activated” (M1) macrophage phenotype and the “alternating-activated” (M2) macrophage phenotype, to achieve a balance between promoting inflammation and inhibiting inflammation in tissues and organs. MSC-derived exosomes can regulate the inflammatory process by promoting M2 macrophage polarization and further balance the excessive inflammation to reduce tissue damage [43, 45, 46].

In this study, we found that TNF-*α*-stimulated MenSCs promoted the production of miR-24-3p in the small EVs. MiR-24-3p in TNF-*α* stimulated MenSC-derived small EVs (MenSCs-sEV ^TNF-*α*^) downregulated the interferon regulatory factor 1 (IRF1) expression and promoted M2 macrophages activation, thus alleviating colonic inflammation.

## 2. Materials and Methods

### 2.1. Animals

All animal experimental operations had been approved by the Ethics Review Committee of the Experimental Animal Center of Zhejiang University, and the ethical approval number is ZJU20210240. For this *in vivo* experiment, male C57BL/6 mice, aged 6–8 weeks, were purchased from the SLAC company (Shanghai, China). All animals were raised in the animal center at 24°C and 60% humidity, with sufficient feed and water. In this study, we strived to reduce the suffering of animals and minimize the number of animals used. In all animal experiments, the number of mice in each group was 6 (*N* = 6).

In the acute colitis model, mice were orally administrated with 5% dextran sulfate sodium (DSS) (YEASEN, China, MW: 36000-50000) for 7 consecutive days, and the clinical symptoms (diarrhea, bloody stool, and weight loss) of mice were monitored by disease activity index (DAI) every day. On day 2 of modeling, mice were injected with small EVs (200 *μ*g) intraperitoneally, and on day 7, 5%DSS will be replaced with ordinary drinking water. On day 9, all acute colitis mice were euthanized to obtain the colon and serum for subsequent experiments.

The methods for chronic modeling were described previously [23]. In the chronic colitis model, the mice were raised with 3% DSS for 1 week, followed by normal water for 1 week, during which time the feed was normal, and then, the above was repeated in one round for a total of 28 days.

After the mice were euthanized, the data of inflammatory infiltration in the colon of the mice were obtained by hematoxylin and eosin (H&E) staining and myeloperoxidase (MPO) activity detection. DAI and H&E staining analysis scoring rules had been reported previously [22]. The scoring rules in this study are shown in Tables 1 and 2.

### 2.2. Intestinal Permeability Test

The intestinal permeability assay in mice is to test intestinal epithelial integrity. The concentration of FITC-dextran was 25 mg/ml, and the dosage of mice was 0.6 mg/g. On day 7 of the acute DSS-colitis model in mice, FITC-dextran was administered to mice by gavage on an empty stomach and mice were sacrificed 4 hours later to obtain the serum. FITC fluorescence can be detected in the mouse serum. Fluorescence intensity is directly proportional to intestinal permeability, with stronger fluorescence indicating more severe intestinal damage.

### 2.3. Isolation and Culture of MenSCs

MenSCs were provided by the Innovative Precision Medicine (IPM) Group, and the specific process of obtaining cells was as above [37, 47, 48]. In brief, menstrual blood samples were collected from healthy young women (*n* = 3) aged 20 to 30 years during menstruation using Divacup (Kitchener, ON). Fresh menstrual blood samples should not be stored for more than 72 hours in a storage solution containing kanamycin sulfate, cefadroxil, vancomycin hydrochloride, amphotericin B, gentamicin sulfate, and heparin in a 4°C refrigerator. Stem cells in menstrual blood were obtained by density gradient centrifugation with Ficoll-Hypaque (DAKEWE, China). The isolated interlayer cells were cultured in Minimum Essential Medium *α* (MEM*α*) (Gibco, USA) adding 15% Australian fetal bovine serum (FBS). MenSCs were completely digested with 0.25% *trypsin*-EDTA (Fisher Scientific, USA) for 5 min, then neutralized with complete medium, and centrifuged to complete subculture. Passages 5-8 (p5-p8) of MenSCs can be used for collection of small EVs.

### 2.4. Identification of MenSCs

To verify the multidirectional differentiation potential of MenSCs, we induced the cells into osteoblastic, adipogenic, and chondrogenic cells for 20 to 30 days [37]. After the differentiation of the cells to a certain extent, they were treated with the corresponding stains and finally photographed with a microscope (Olympus, Japan). The surface molecules of MenSCs were detected by flow cytometry (ACEA NovoCyte, ACEA Biosciences, USA).

### 2.5. The Isolation of MenSC-Derived Small EVs

As mentioned above, small EVs were isolated from the cell culture supernatants of MenSCs by ultracentrifugation [38]. After the passage of MenSCs to P5-P8, the cells adhered to the wall overnight and were cultured with or without 20 ng/ml TNF-*α* to 80%–90% fusion degree. Then, the MenSCs were cultured in a pure MEM*α* medium for 48 h. The collected cell supernatants of MenSCs were subjected to low-speed centrifugation several times, 300 g for 10 minutes, 2000 g for 20 minutes, and 10000 g for 30 minutes. During the above centrifugation process, the precipitate was discarded; in other words, dead cells and cell debris in the supernatant were removed. Then, the supernatant obtained in the previous step was filtered with a 0.22 *μ*m filter (Millipore, USA). The filtrate was centrifuged twice at 100,000 g for 70 min and finally obtained the small EVs. For the following Western blotting assays of small EVs, the protein in exosomes was lysed with 50 *μ*l RIPA lysate (Beyotime Biotechnology, China), and the protein was quantified with a BCA kit (Beyotime Biotechnology, China). The microRNA in small EVs was isolated by microRNA extraction kit (Qiagen, USA), and the relative expression of miR-24-3p was measured by quantitative polymerase chain reaction (q-PCR).

### 2.6. Identification of MenSC-Derived Small EVs

The small EVs were identified by transmission electron microscopy (TEM) (Thermo FEI, Czech Republic) observation, nanoparticle tracking analysis, and Western blotting. For TEM identification, small EVs were first fixed with 2% paraformaldehyde solution and then 5 *μ*l of EV suspension was added to the Formvar-carbon copper grid. After draining excess liquid from the copper mesh, it was stained with 1% uranyl acetate. Immediately afterward, the copper mesh was allowed to stand for 5 minutes at room temperature and then washed with deionized distilled water. After drying, the sample was observed under the transmission electron microscope. The particle size distribution and concentration of small EVs were tested by Nanosight NS 500 (malvin, England). After lysing MenSCs and small EVs with RIPA lysate, the amount of protein was corrected with a BCA kit to ensure that the quality of each group of samples was 10 *μ*g. The Western blotting experiments of GAPDH, CD63, CD81, and TSG101 were then carried out.

### 2.7. Internalization of MenSC-Derived Small EVs

By labeling small EVs *in vitro* to clarify the location of small EVs in macrophages, we further explored the possible mechanisms of small EV action. MenSCs-derived small EVs (MenSCs-sEV) and MenSCs-sEV^TNF-*α*^ were stained with red fluorescent dye PKH26 (Sigma-Aldrich, USA), as described above [49]. Briefly, we added 1 *μ*l PKH26 to 50 *μ*l Dilution C and 10 *μ*g exosomes to 50 *μ*l Dilution C in another tube. They were then mixed and allowed to stand at room temperature for 5 min. The staining was then terminated using 500 *μ*l of EVs-depleted FBS, and the small EVs were isolated again by ultracentrifugation. The mouse leukemic monocyte/macrophage cell line RAW264.7 was cultured on a dedicated cell slide for 24-well plates and PKH26-labeled small EVs were cocultured with the cells when they reached 70% fusion. After 48 hours, the cells were fixed with a 4% paraformaldehyde solution and stained with FITC-phalloidin and DAPI, respectively. The slides were sealed with an antifluorescence quenching sealing tablet and observed with an FV3000-OSR microscope (Olympus, Japan).

### 2.8. Cell Transfection

Mimics and inhibitors of miR-24-3p were synthesized by RiboBio Corporation (Guangzhou, China). IRF1 small interfering RNAs (siRNAs) and IRF1 overexpressed plasmids were synthesized by Sangon Biotech Corporation (Shanghai, China). The Lipofectamine™ 3000 transfection reagent (Thermo Fisher, USA) was used to transfect plasmids, microRNA mimics, microRNA inhibitors, and siRNAs. The specific steps were following the instructions provided. The transfection efficiency was measured by qPCR or Western blotting.

### 2.9. Dual-Luciferase Reporter Gene Assay

The wild-type (WT) and mutant-type (MUT) 3′-UTR of IRF1 mRNA were synthesized by Oligobio Biotech Corporation (Beijing, China). This corporation also synthesized the recombinant pmirGLO plasmid containing the WT sequence or MUT sequence. Human embryonic kidney cells (HEK293T, ATCC) were cultured in an incubator at 37°C with 12-well plates. When the fusion degree reached 60%, 1 *μ*g/ml plasmids and 50 nM miR-24-3p mimics were cotransfected with serum-free Dulbecco's modified Eagle medium (DMEM) (Thermo Fisher, USA) and the Lipofectamine™ 3000 transfection reagent. After 48 h, cells were lysed with lysate from the luciferase reporter gene assay kit (Promega, USA). According to the instructions, the cell lysates were transferred to a white plate. After the addition of LAR II and Stop & Glo reagents (Thermo, USA), the substances in the well plates were quantified twice by the microplate reader and calculated.

### 2.10. Quantitative PCR and Western Blotting

Total RNA isolation and purification of Raw264.7 cells were performed with the aid of the RNeasy mini kit (Qiagen, USA), and all operations were completed according to the kit instructions (Qiagen, USA). Quantitative analysis was accomplished using the -2^*ΔΔ*Ct^ method [50]. All primers (including miRNA) used for quantitative real-time PCR are presented in Table S1. As previously described, the specific experimental operations of reverse transcription, qPCR, protein extraction, and Western blotting were performed [37, 39]. Before Western blotting assays, the protein concentrations were measured using the BCA kit (Beyotime, China) to equalize the protein concentrations among the groups. Then, 4× loading buffer was added to the protein and they were boiled in a metal bath at 100°C for 10 min. Finally, the ECL luminescent solution (Bio-Rad, USA) was dropped on the PVDF membrane, and the band of protein could be observed by the ChemiDoc Touch Imaging System (Bio-Rad, USA).

### 2.11. Enzyme-Linked Immunosorbent Assay (ELISA) for Cytokine Analysis

The distal colon of each group was weighed and placed in liquid nitrogen for rapid cryopreservation. The colon samples were thoroughly ground in a homogenizer, and the supernatant of tissue homogenate was collected after centrifugation. The levels of TNF-*α*, interleukin(IL)-6, IL-10, IL-1*β,* and IFN-*γ* in the colonic tissues of mice were measured by the ELISA kit (FANKEW, China). The specific steps of the assay were carried out following the manufacturer's instructions. The absorbance of the sample and standard wells in the ELISA plate was detected at 450 nm with the microplate reader (Biorad, USA).

### 2.12. Isolation of Mononuclear Cells from Lamina Propria of the Colon

After the mice were euthanized, the colon was removed and cut into 1 cm segments. The epithelial digestive fluid (HBSS+5 mM EDTA+1 mM DTT), lamina propria digestive fluid (Collagenase D+DNase I+Dispase II+ PBS), 40%/80% Percoll were prepared in advance. The segments of the colon were first digested several times with epithelial digestive fluid, and they were digested again with lamina propria digestive fluid after the epithelial cells were discarded. Colon tissue fragments from the lamina collected after digestion were filtered through a 40 *μ*m cell sieve, and all obtained cells were subjected to a density gradient centrifugation of 40%/80% Percoll. Further, the white interlaminar cells were collected and centrifuged, and the cell viability should be greater than 95% by the Trypan blue test.

### 2.13. Clearance of Colon Macrophages

To investigate the role of macrophages in MenSCs-sEV^TNF-*α*^ in IBD mice, we used chlorophosphate liposomes (Clop-lipo) (Formumax, USA) to remove macrophages in animal experiments. Two days before DSS was added to the drinking water of mice, 200 *μ*l Clop-lipo was intraperitoneally injected into mice, and the same dose of PBS-lipo was used as a control. After one week, the proportion of macrophages in lamina propria mononuclear cells was measured by flow cytometry.

### 2.14. Immunohistochemistry (IHC) Analysis

In order to explore the effect of different treatments on the integrity of mouse colon epithelium, IHC staining of zonula occludens- (ZO-) 1, ZO-2, and occludin was performed on every mouse colon. The colon tissues were formalin-fixed and paraffin-embedded. Then, they were cut into 5 *μ*m paraffin sections with a paraffin slicing machine. After the sections were placed in 0.01 mol/l (PH7.8) citric acid buffer for antigen repair, antibodies against ZO-1, ZO-2, and occludin were diluted according to the instructions and then dropped onto the sections for overnight incubation. In the final step, second antibodies was added the next day; then, the sections were mounted with resin after diaminobenzidine and hematoxylin staining.

### 2.15. Statistics

GraphPad Prism 8.0.2 (San Diego, CA) was used to statistically analyze the data differences between groups. In Graphpad Prism software, the Agostino-Pearson method was used to detect the normality of experimental data. When *p* > 0.1, the data is normally distributed. The data in this study conform to a random and normal distribution with uniform variance. Therefore, the *t*-test was used for comparison between the two groups. One-way ANOVA was used for multigroup comparison, and both of them are parametric tests. A *p* value lower than 0.05 was considered as a significant difference, which means that the data was meaningful. ^∗^*p* < 0.05, ^∗∗^*p* < 0.01, and ^∗∗∗^*p* < 0.001.

## 3. Results

### 3.1. Identification of MenSCs and Small EVs

**Figure 1:**
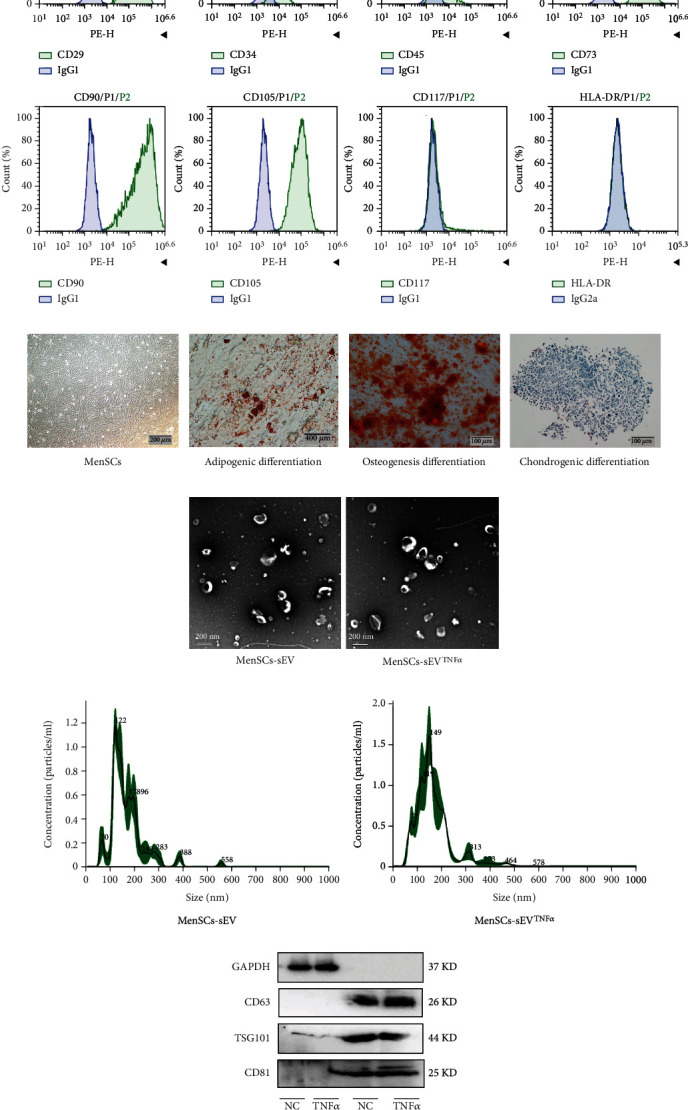
The characterization of MenSCs and MenSC-derived small EVs. (a) The expression of MenSC surface molecules was detected by flow cytometry. Among them, IgG1 antibody is the isotype control of CD29, CD34, CD45, CD73, CD90, CD105, and CD117, and IgG2a is the isotype control of HLA-DR. (b) Optical images of the trilineage differentiation staining and morphology of MenSCs. (c) Transmission electron micrographs of small EVs. (d) Nanoparticle tracking analysis (NTA) of MenSCs-sEV and MenSCs-sEV^TNF-*α*^. (e) Western blotting was used to measure the expression of CD63, TSG101, and CD81 molecules on the surface of MenSCs and their small EVs.

MenSCs were identified by optical images of cells, surface phenotype, and trilineage differentiation staining. The results of flow cytometry showed that CD29, CD73, CD90, and CD105 molecules were highly expressed on the surface of MenSCs, while HLA-DR, CD34, CD45, and CD117 molecules were extremely low or almost absent (Figure 1(a)). Microscopically, MenSCs appeared spindle-shaped and fibrous (Figure 1(b)). The pluripotency of MenSCs can be evaluated by the tri-lineage differentiation assay. When MenSCs were cultured in a specific induction medium for 21~30 days, alizarin red staining-positive (osteogenic differentiation), oil red O-stained lipid droplets (adipogenic differentiation), and alcian blue-stained chondrocytes (chondrogenic differentiation) could be observed (Figure 1(b)). Through TEM, the morphology of MenSCs-sEV and MenSCs-sEV^TNF-*α*^ can be visually seen, which are oval bilayer lipid vesicles less than 200 nm in diameter (Figure 1(c)). In the nanoparticle tracking analysis, the mean diameter of MenSCs-sEV was 166.8 nm, while the mean diameter of MenSCs-sEV^TNF-*α*^ was 163.9 nm (Figure 1(d)). In Western blotting, CD63, CD81, and TSG101 showed clear bands in MenSCs-sEV and MenSCs-sEV^TNF-*α*^, while these molecules showed weak or no bands in their corresponding MSCs (Figure 1(e)).

### 3.2. TNF-*α*-Pretreated MenSC-Derived Small EVs Alleviated DSS-Induced Acute IBD

**Figure 2:**
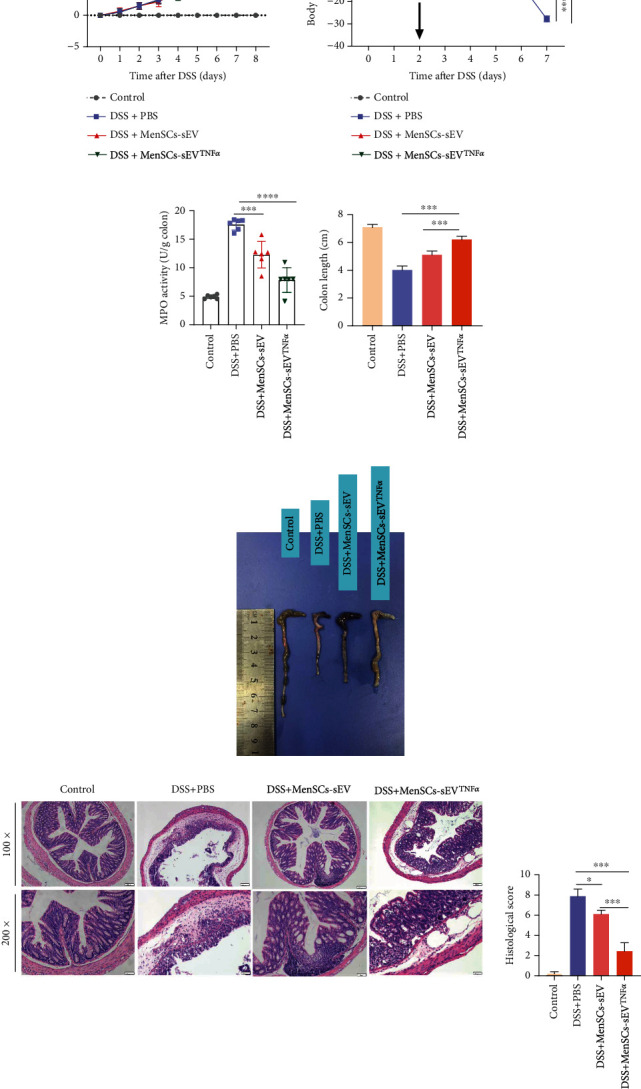
TNF-*α*-pretreated MenSC-derived small EVs can relieve colonic inflammation induced by DSS. Male wild-type C57BL/6 mice aged 6-8 weeks were given 5% DSS for one week and intraperitoneally injected with MenSCs-sEV and MenSCs-sEV^TNF-*α*^ (200 *μ*g/mouse) (*n* = 6) on day 2. (a, b) DAI scores and the weight of the mice were recorded daily. (c) Neutrophil infiltration in the colon can be measured with the MPO activity detection kit. (d, e) On day 8, after the mice were euthanized, colon length data and gross colon images were obtained. (f) After soaking in 4% paraformaldehyde for 48 hours, the colon was dehydrated and stained with hematoxylin and eosin. Original magnification: ×100 (up) and ×200 (down).

On the day following oral administration of 5% DSS, mice developed diarrhea, and the intraperitoneal injection of small EVs can alleviate the subsequent clinical signs compared to the DSS+PBS group. The clinical symptoms of the mice were recorded as DAI. Statistics were made to visualize the severity of the disease, in which we found that the efficacy of MenSCs-sEV^TNF-*α*^ was better than MenSCs-sEV (Figure 2(a)). Daily body weight monitoring showed that weight loss in all groups except the control group, and the loss of weight in the DSS+MenSCs-sEV^TNF-*α*^ group was less than that in the other groups (Figure 2(b)). Excessive neutrophils can also damage the tissue. The MPO activity was used to reflect the level of neutrophil infiltration. After small EV therapy, we found that MPO activity decreased (Figure 2(c)) and colon length did not shorten as much as the untreated group (Figure 2(d)). As can be seen from the direct view of the colon, the overall colon of the DSS+PBS group was red, with increased permeability, thin, easily damaged, loosen colon contents, and severe shortening, while the small EV-treated groups, including MenSCs-sEV and MenSCs-sEV^TNF-*α*^ group, had better morphology (Figure 2(e)). H&E staining showed that DSS caused incomplete colonic structure, loss of crypt, structural destruction, and infiltration of inflammatory cells. Compared with the DSS+PBS group and the DSS+MenSCs-sEV group, the DSS+MenSCs-sEV^TNF-*α*^ group had less damage and fewer leukocytes in the colon tissue (Figure 2(f)). Further, we conducted a semiquantitative analysis of the results of H&E staining with a double-blind situation, and the results were presented by pathological scores (Figure 2(f)). The degree of colonic damage is positively correlated with the pathological score. After colitis mice were treated with small EVs, the pathological scores of the murine colon decreased significantly. Given the above, MenSC-derived small EVs can alleviate colonic inflammation in mice, and the efficacy of MenSCs-sEV^TNF-*α*^ was superior to MenSCs-sEV.

### 3.3. MenSCs-sEV^TNF-*α*^ Can Affect the Colonic Inflammatory Cytokines and Intestinal Epithelial Integrity

**Figure 3:**
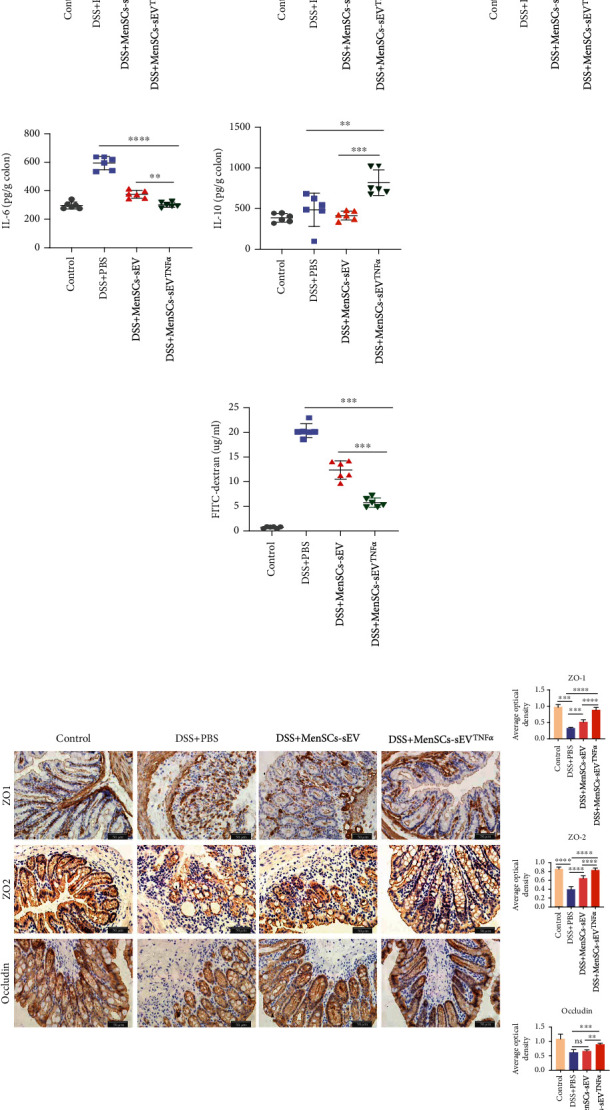
MenSCs-sEV^TNF-*α*^ reduces the expression of proinflammatory cytokines and promotes the maintenance of intestinal integrity in IBD mice. (a) The levels of cytokines in the mouse colon were detected by ELISA (*n* = 6). (b) Mice were gavaged with FITC-dextran (0.6 mg/g body weight) under fasted conditions and FITC fluorescence intensity in serum was detected 4 hours later (*n* = 6). (c) IHC was used to obtain optical images of the distribution and content of tight junction proteins ZO-1, ZO-2, and occludin. Original magnification, ×400. ImageJ was used to quantify the tight junction proteins content in immunohistochemical images.

Disorders of inflammatory mediators and intestinal barrier dysfunction are two salient characteristics of IBD [51–53]. In the ELISA results, the expression of TNF-*α*, IFN-*γ*, IL-1*β*, and IL-6 was downregulated and IL-10 expression was upregulated in the mouse colon after intraperitoneal injection of MenSCs-sEV or MenSCs-sEV^TNF-*α*^ (Figure 3(a)). To explore the integrity of intestinal epithelium, we investigated the expressions of the colonic tight junction proteins ZO-1, ZO-2, and occludin using immunohistochemistry, as well as the intestinal permeability test. During the intestinal permeability test, the fluorescence intensity of FITC in the serum reflects the amount of dextran in the serum that infiltrates from the colon. High intestinal permeability indicates that after injury the intestinal epithelium is not intact. We found that the FITC fluorescence value in the serum of mice of the control group was the minimum and that in the DSS+MenSCs-sEV^TNF-*α*^ group was significantly less than the other two groups (Figure 3(b)). Further, we detected the content and distribution of tight junction proteins ZO-1, ZO-2, and occludin in the colon of mice by immunohistochemistry assays. These three tight junction proteins were distributed more in the colon of the control group and DSS+MenSCs-sEV^TNF-*α*^ group, while the other two groups were less. The expression of ZO-1 and ZO-2 in DSS+MenSCs-sEV group was significantly higher than that in the DSS+PBS group, but there was no difference in the occludin expression between the two groups (Figure 3(c)).

### 3.4. MenSCs-sEV^TNF-*α*^ Alleviated DSS-Induced Chronic IBD In Vivo

**Figure 4:**
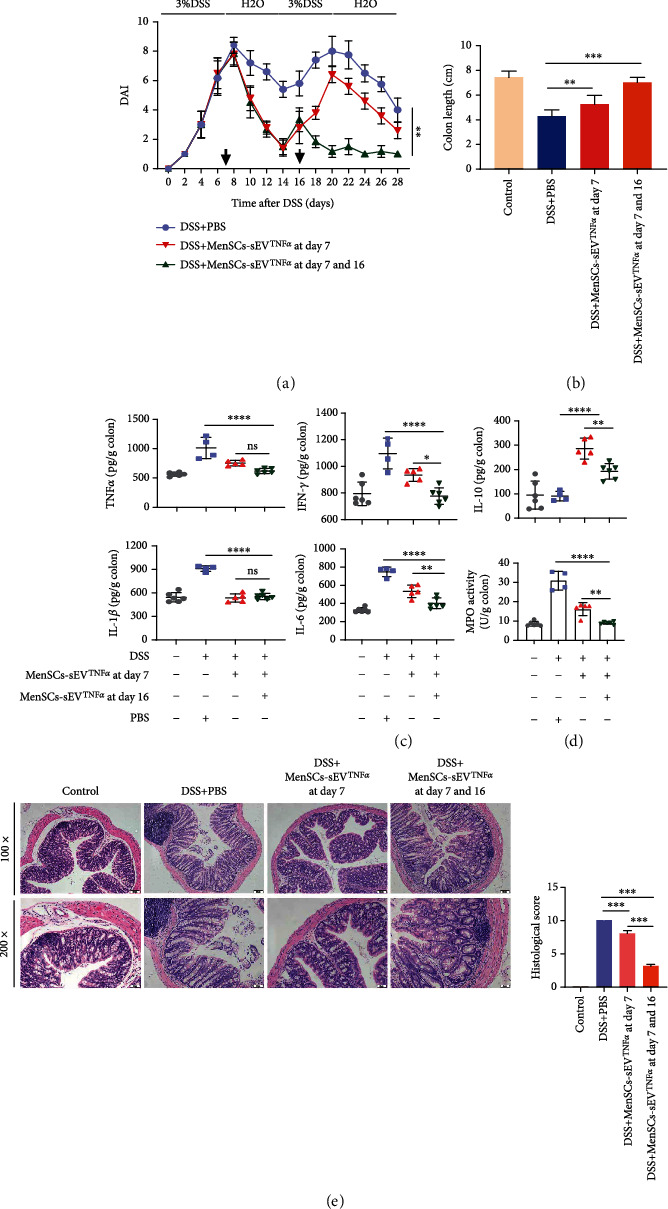
MenSCs-sEV^TNF-*α*^ relieves chronic recurrent colitis caused by DSS. Male C57 BL/6 mice (*n* = 6) aged 6-8 weeks were given normal water for 7 days after 3% DSS solution for 1 week. The treatment was repeated once and small EVs were intraperitoneally injected into the DSS+MenSCs-sEV^TNF-*α*^ group on day 7 and day 16 (200 *μ*g/mouse). (a) The clinical symptoms of the mice were recorded daily as DAI scores, which were based on the varying degrees of weight loss, stool consistency, and hematochezia. (b) The length of the colon of each mouse was measured after euthanasia. (c) The levels of TNF-*α*, IFN-*γ*, IL-10, IL-1*β*, and IL-6 in colon tissue were measured by ELISA. (d) Neutrophil infiltration in the colon was detected by MPO activity. (e) Colon tissues were stained with hematoxylin and eosin to observe the inflammatory infiltration and structural integrity. Original magnification: ×100 (up) and ×200 (down).

To explore the role of MenSCs-sEV ^TNF-*α*^ in chronic and recurrent IBD, we orally administered 3% DSS to mice for two weeks discontinuously. And we found that mice in each group experienced repeated hematochezia, diarrhea, and weight loss. Mice injected with small EVs twice (day 7 and 16) had better clinical performance (Figure 4(a)). In addition, the colon length of mice with MenSCs-sEV^TNF-*α*^ injections on day 7 and day 16 was not significantly shortened, but that of mice without injection and only injecting once was clearly shortened (Figure 4(b)). ELISA showed that the levels of TNF-*α*, IFN-*γ*, IL-1*β*, and IL-6 were decreased in the mouse colon with intraperitoneal injections of MenSCs-sEV^TNF-*α*^ on both day 7 and day 16. Unexpectedly, the IL-10 level appeared to be higher in a single dose than in two (Figure 4(c)). In addition, the MPO activity of the mouse colon was also detected in the same way as the acute modeling process. After two rounds of treatment, the degree of neutrophil infiltration in the colon was slight, which was consistent with ELISA results (Figure 4(d)). In all animal experiments, the number of mice in each group was 6 (*N* = 6) at first. However, in the process of chronic modeling, because of the strong toxicity of DSS, two mice in the DSS group were euthanized in the experiment for poor conditions. In addition, one mouse in the DSS+MenSCs-sEV^TNF-*α*^ at day 7 group died during the second disease peak due to intolerance to DSS.

H&E staining indicated that the colon epithelium of the two-dose mice was intact, with normal crypt and goblet cell structure and only a small number of inflammatory cell infiltration. In the colon of mice treated with small EVs only on day 7, fossa damage and inflammatory cells infiltration were evident (Figure 4(e)). In general, the mice showed a course of exacerbation, remission, and relapse during chronic IBD modeling. The efficacy of injecting twice during IBD exacerbation and relapse was better than injecting once.

### 3.5. MenSCs-sEV^TNF-*α*^ Increased the Proportion of M2 Phenotype of Macrophages In Vitro

**Figure 5:**
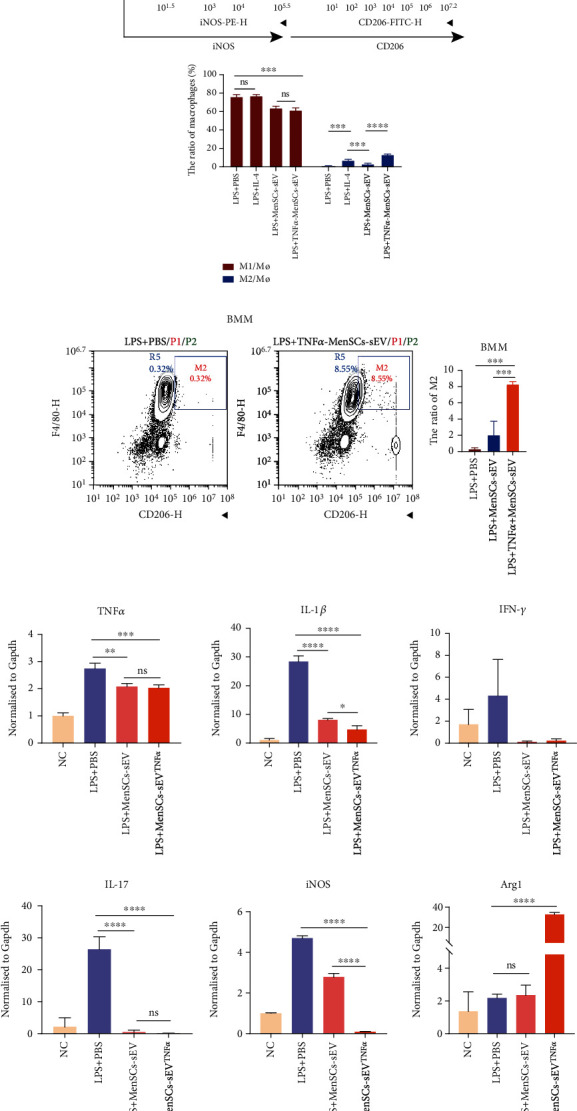
MenSCs-sEV^TNF-*α*^ converts M1 macrophages into M2 macrophages in vitro. (a) Raw264.7 cells were treated with 100 ng/ml LPS and 100 ng/ml IL-4 or 100 *μ*g/ml small EVs to measure the ratio of M2 by flow cytometry. IL-4 was used as a positive control. (b) The level of macrophage polarization was measured in murine bone marrow-derived macrophage (BMM) treated with MenSCs-sEV (100 *μ*g/ml) or MenSCs-sEV^TNF-*α*^ (100 *μ*g/ml) by flow cytometry. (c) The expression of TNF-*α*, IL-1*β*, IL-17, IFN-*γ*, iNOS, and Arg1 of RAW264.7 after different treatments were detected by PCR. (d) PKH26-labeled MenSCs-sEV and MenSCs-sEV^TNF-*α*^ were absorbed by RAW264.7 cells, which were stained with FITC-phalloidine and DAPI and observed with confocal microscopy.

The small EV intraperitoneal injection caused variation in the expression of inflammatory cytokines in the colon of mice. And these inflammatory cytokines, which are mainly secreted by macrophages, are key factors mediating the progression of IBD. As a result, MenSCs-sEV^TNF-*α*^ may alleviate colonic inflammation by altering macrophage functions. To study whether MenSCs-sEV^TNF-*α*^ could promote the polarization of M2 macrophages *in vitro*, MenSCs-sEV^TNF-*α*^ (100 *μ*g/ml) was added to Raw 264.7 cell culture. First, Raw 264.7 cells were stimulated with 100 ng/ml LPS to expose macrophages to an inflammatory microenvironment. After LPS stimulation, Raw264.7 cells were mainly M1 macrophages, while the number of M2 macrophages accounted for less than 2%. Then after 48 hours of coculture with MenSCs-sEV^TNF-*α*^, flow cytometry showed that the proportion of CD206^+^ cells (M2 macrophages) exceeded 10% (Figure 5(a)). To some extent, cell lines may not fully represent the reality of living cells in the tissue. In order to enhance the credibility of the results, we isolated mouse bone marrow-derived macrophages (BMM), then treated them with LPS and MenSCs-sEV^TNF-*α*^, and finally obtained similar results (Figure 5(b)). To further explore the levels of inflammatory factors under different treatments, we detected the relative expression levels of TNF-*α*, IL-1*β*, IFN-*γ*, IL-17, iNOS, and Arg-1 in Raw264.7 cells by qPCR. Compared with the LPS+PBS group, MenSCs-sEV^TNF-*α*^ and MenSCs-sEV decreased the levels of proinflammatory cytokines in Raw264.7 cells, and IL-1*β* and iNOS expressions in the MenSCs-sEV^TNF-*α*^ group were lower than those in the MenSCs-sEV-treated group (Figure 5(c)). In Raw264.7 macrophages, MenSCs-sEV^TNF-*α*^ can downregulate the expression of iNOS and upregulate Arg1, indicating that MenSCs-sEV^TNF-*α*^ may convert M1 macrophages into M2 macrophages. To further figure out how small EVs affect macrophages, we labeled small EVs with PKH26 and observed the position of the red fluorescence of PKH26 under the confocal microscope. PKH26 red dots could be seen in the cytoplasm of macrophages by immunofluorescence staining after PKH26-labeled small EVs treating macrophages (Figure 5(d)). It is concluded above that small EVs were located in the cytoplasm after phagocytosis by macrophages, furtherly altering the secretion of inflammatory cytokines in macrophages.

### 3.6. MenSCs-sEV^TNF-*α*^ Elevates the Ratio of M2 Macrophages in the Colon of Mice with DSS-Colitis

**Figure 6:**
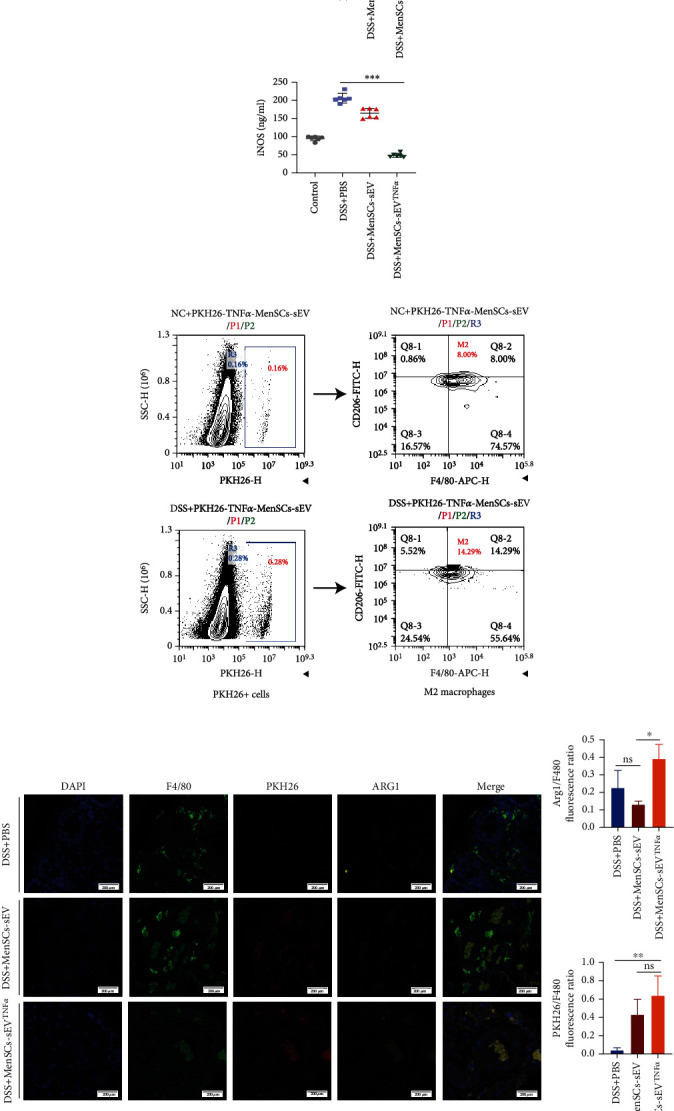
MenSCs-sEV^TNF-*α*^ can increase the proportion of M2 macrophages in vivo. (a) The level of Arg1 and iNOS in the colon of mice was measured by ELISA. (b) Flow cytometry showed the proportion of PKH26+ cells (absorbing PKH26-labeled MenSCs-sEV^TNF-*α*^) and M2 macrophages in colonic lamina propria mononuclear cells. (c) PKH26-labeled small EVs were intraperitoneally injected into mice, and the immunofluorescence staining of DAPI, F4/80, and Arg1 in the colon of mice was shown in the confocal microscope.

We had previously proved that MenSCs-sEV^TNF-*α*^ can increase the number of M2 macrophages *in vitro* and further explored whether the same phenomenon can be observed *in vivo*. In a mouse model of acute IBD, the colons of euthanized mice at day 9 were removed to perform ELISA for iNOS and Arg-1. The results showed that Arg-1 expression was upregulated and iNOS expression was downregulated in the colon of mice treated with MenSCs-sEV^TNF-*α*^ (Figure 6(a)). Flow cytometry showed that after oral DSS treatment, the proportion of PKH26^+^ mononuclear cells and M2 macrophages in the lamina propria was dramatically higher than that in the NC group (Figure 6(b)). In the immunofluorescence assay of colonic tissue, the relative fluorescence intensity of Arg-1 of the MenSCs-sEV^TNF-*α*^ group were obviously higher than other two groups, and the positive sites of Arg1 were identical to PKH26 (Figure 6(c)). This demonstrated that macrophages that absorbed MenSCs-sEV^TNF-*α*^ could be converted into M2 macrophages.

Both *in vivo* and *in vitro*, MenSCs-sEV^TNF-*α*^ can cause significant changes in macrophages, so we speculated that MenSCs-sEV^TNF-*α*^ alleviates colonic inflammation mainly through macrophages. To verify the important role of macrophages in this process, we tried to eliminate macrophages in mice with chlorophosphate liposomes (clop-lipo) (FormuMax, USA) in an acute IBD mouse model. After a one-time intraperitoneal injection of 250 *μ*l clop-lipo, cells from the bone marrow of mice were collected and detected by flow cytometry 7 days later. The results of flow cytometry showed that the ratio of F4/80^+^ cells decreased by about 80%, which proved that clop-lipo successfully eliminated most macrophages in mice (Figure S1E). In the acute IBD model, MenSCs-sEV^TNF-*α*^ could not affect the colitis phenotype after intraperitoneal injection of clop-lipo (Figure S1A, B, C, D).

### 3.7. TNF-*α* Induces Changes in MicroRNAs in Small EVs Derived from MenSCs

**Figure 7:**
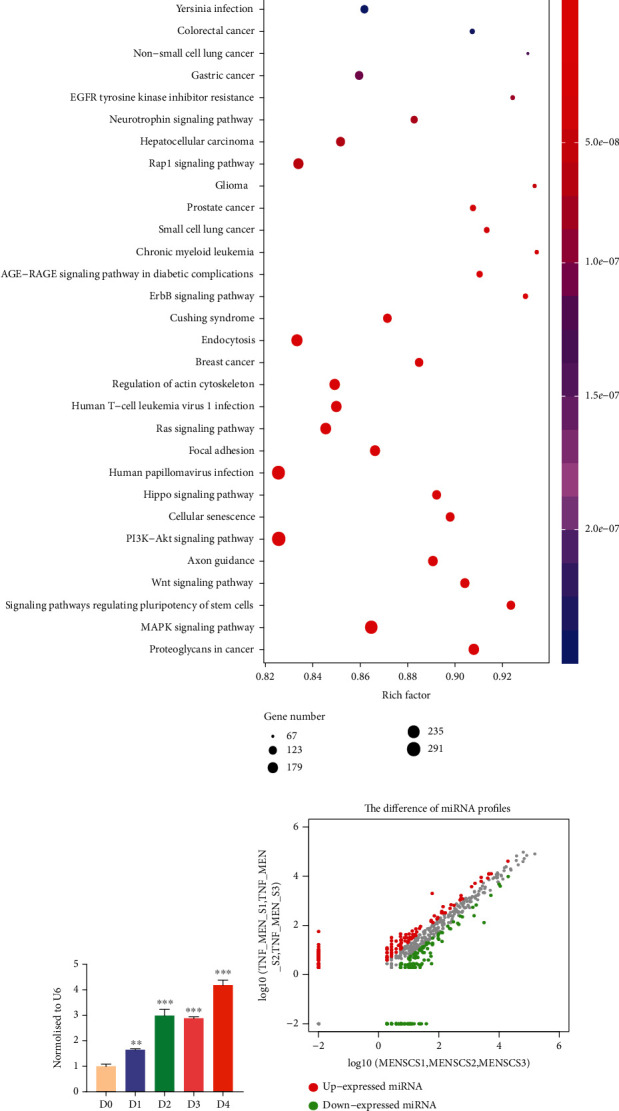
Expression difference analysis of microRNA in MenSCs-sEV and MenSCs-sEV^TNF-*α*^. (a) Heat map of microRNA expression difference between MenSCs-sEV and MenSCs-sEV^TNF-*α*^ of microRNA sequence. (b) KEGG was used to analyze the signaling pathways involved in the differential expression of microRNA between MenSCs-sEV and MenSCs-sEV^TNF-*α*^. (c) MicroRNA sequencing results were verified by qPCR. After 0, 1, 2, 3, and 4 days of TNF-*α* pretreatment, the expression value of miR-24-3p relative to u6 increased with the pretreatment days. (d, e) The difference of microRNA expression between MenSCs-sEV^TNF-*α*^ and MenSCs-sEV was presented by the volcano map and microRNA scatter map. (f) The viability of MenSCs treated with TNF-*α* of different concentrations was detected by the CCK8 kit.

Sequencing of MenSCs-sEV and MenSCs-sEV^TNF-*α*^ was performed on Illumina HiSeqTM 2500 platform by Guangdong RiboBio Biotechnology Co. Ltd. The differential expression results of sequencing were presented in Table S2. Functional heatmaps were used to show microRNA expression differences through hierarchical clustering analysis. Through the difference ratio (|log2(Fold Change)| > 1) and the significant level (*p* value < 0.05) of these two indicators, we picked out the differential expression microRNAs. Hsa-miR-708-5p, hsa-miR-24-3p, and so on were significantly upregulated in exosomes secreted by MenSCs after TNF-*α* stimulating, while hsa-miR-365a-5p, hsa-miR-490-5p, and so on were significantly downregulated (Figure 7(a)). Differential microRNA expression was reflected by microRNA differential expression scatterplot and microRNA differential expression volcano map between samples: there were 70 differential microRNAs, 38 of which were upregulated and 32 downregulated (Figures 7(d) and 7(e)). Enrichment analysis showed that the differential expression of microRNAs was related to specific viral and bacterial infection, endocytosis, PI3K-AKT signaling pathways, and signaling pathways that regulate stem cell pluripotency (Figure 7(b)). TNF-*α* changed the function of small EVs of MenSCs, and differentially expressed microRNAs from TNF-*α*-MenSCs participated in different biological processes. Through the analysis of differential microRNAs and their downstream target proteins, we found that only the downstream of miR-24-3p was closely related to inflammation relief and macrophage function alterations, so we only made further research on miR-24-3p.

In order to verify the sequencing results, we extracted the small EVs from the supernatants of MenSCs which were treated with TNF-*α* for 0, 1, 2, 3, and 4 days and used qPCR to obtain the relative expression of miR-24-3p. The results showed that the longer the MenSCs were exposed to TNF-*α*, the higher the content of miR-24-3p in MenSCs-sEV^TNF-*α*^ would be (Figure 7(c)). TNF-*α* concentration can affect the cell viability of MenSCs. MenSCs treated with different concentrations of TNF-*α* were quantified by Cell Counting Kit 8 (CCK8). After being treated with 50 ng/ml TNF-*α* for 48 h, the number of MenSCs was significantly decreased (Figure 7(f)). Hence, 20 ng/ml TNF-*α* is a safe concentration for promoting MenSCs to secret anti-inflammatory small EVs.

### 3.8. MiR-24-3p in MenSCs-sEV^TNF-*α*^ Converts M1 to M2 by Downregulating IRF1

**Figure 8:**
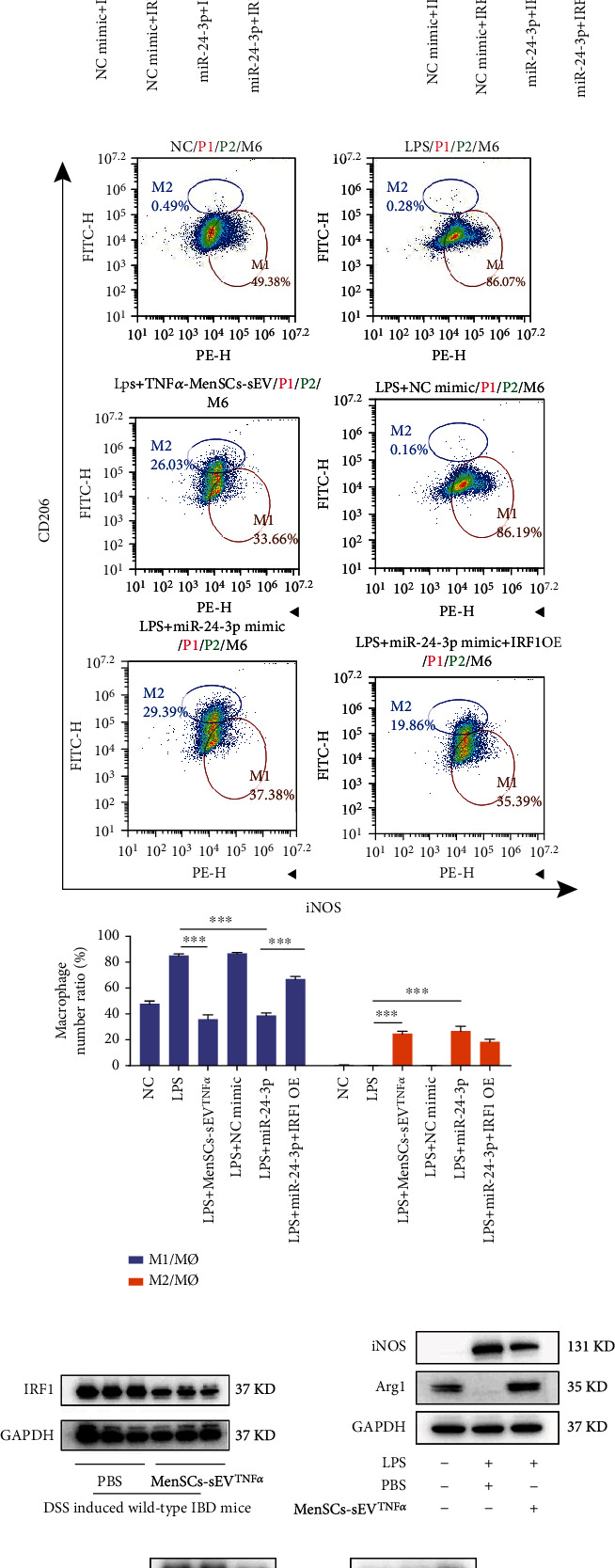
MenSCs-sEV^TNF-*α*^ promotes the polarization of M2 macrophages through miR-24-3p and its downstream IRF1. (a) Luciferase reporter gene assay showed that miR-24-3p could bind to site 1 of IRF1. (b) Representative flow cytometry images showed the proportion of M1(F4/80+iNOS+) and M2(F4/80+CD206+) macrophages in RAW264.7 cells with different treatments. (c) The protein expression of IRF1 was presented by Western blotting after intraperitoneal injection of MenSCs-sEV^TNF-*α*^ in DSS-induced acute IBD mice. (d) Western blotting images of RAW264.7 with three treatments, including control, LPS+PBS, and LPS+MenSCs-sEV^TNF-*α*^. (e) The effects of si-IRF1 and IRF1 OE plasmid transfection on the expression of IRF1 in RAW264.7 were verified by Western blotting. (f) After processing RAW264.7 with LPS and knockdown or overexpression (OE) of miR-24-3p and IRF1, the Western blotting image of iNOS and Arg1 was obtained.

Previous studies had shown that IRF1 was closely related to macrophage polarization [54–57]. Moreover, the website TargetScan Human 7.1 predicted that there might be two binding sites between IRF1 and miR-24-3p. In the presence or absence of miR-24-3p overexpression (OE), the WT and MUT 3′-UTR of IRF1 site 1 were used for luciferase reporter gene detection. The binding of miR-24-3p with 3′-UTR WT resulted in the decrease of luciferase activity, while the 3′-UTR MUT could not bind to miR-24-3p, the luciferase activity remained unchanged. It proved that site 1 was the only binding site between miR-24-3p and IRF1 (Figure 8(a)). *In vivo*, intraperitoneal injection of MenSCs-sEV^TNF-*α*^ downregulated the expression of IRF1 in the colon of mice, which was verified by western blotting (Figure 8(c)). *In vitro*, the Western blotting assay also confirmed that MenSCs-sEV^TNF-*α*^ can make M1 marker iNOS downregulate and M2 marker Arg1 upregulate in RAW264.7 cells (Figure 8(d)).

The mechanism of MenSCs-sEV^TNF-*α*^ transforming macrophages from M1 to M2 was demonstrated by flow cytometry and Western blotting analysis *in vitro*. When flow cytometry was performed on Raw264.7 cells with different treatments, we found that LPS stimulation significantly increased the proportion of M1 macrophages, while MenSCs-sEV^TNF-*α*^ addition decreased the number of M1 and increased M2 proportion. The effect of MenSCs-sEV^TNF-*α*^ in RAW264.7 was the same as that of the miR-24-3p mimic, both of which could increase M2 macrophages by more than 20%. We found that OE of IRF1 counteracted the macrophage polarization-promoting effect of miR-24-3p, which demonstrates that miR-24-3p worked only by binding IRF1 mRNA in Raw264.7 (Figure 8(b)). In the Western blotting assay, when MenSCs-sEV^TNF-*α*^ was added to Raw264.7 or miR-24-3p mimic was transfected, the M2 marker was upregulated and the M1 was downregulated. When microRNA was overexpressed (miR-24-3p mimic transfection), the downstream target protein IRF1 was also decreased, and vice versa. Knockdown (si-IRF1) or OE of IRF1 can partially rescue the effects of miR-24-3p mimic or inhibitor. In addition, after miR-24-3p was inhibited, MenSCs-sEV^TNF-*α*^ would no longer promote the polarization of macrophages (Figure 8(f)). In general, miR-24-3p affected M2 production by interfering with the expression IRF1. The influence of si-IRF1 and IRF1 OE on the expression of IRF1 was verified by Western blotting (Figure 8(e)).

## 4. Discussion

Menstrual blood, as a kind of metabolic waste of the female body, can cure illnesses because it contains MenSCs. In this study, MenSCs were derived from menstruating women between 20 and 30 years old. The previous report showed that the cellular status of MenSCs changes with the age of donors. MenSCs from older donors (>40 years old) have weak long-term passage ability. EVs of MSCs derived from the bone marrow and umbilical cord had been proven to have alleviating effects on IBD. Exosomes of MSCs from the bone marrow relieved IBD through macrophage, which is related to metallothionein-2 in exosomes [23]. The exosomes of umbilical cord-derived MSCs cured IBD through microRNA, which is related to miR-326 and the NF-*κ*B pathway [58]. The functions and characteristics of MSC and their secreted EVs from different sources (the bone marrow, adipose, umbilical cords, etc.) are generally the same, but there are some differences in the therapeutic mechanisms for diseases [59]. Since MenSCs-sEV was not as effective as MenSCs-sEV^TNF-*α*^ on DSS-colitis, the mechanism of MenSCs-sEV^TNF-*α*^ in alleviating colitis was mainly investigated in this study. MenSCs are a new type of MSCs with abundant sources. The application of MenSCs in the treatment of IBD and its possible mechanism are the highlights of this study. The weakness of this study is that we focused too much on macrophages and ignored the possible role of other immune-related cells, such as lymphocytes, colonic epithelial cells, and neutrophils. We need to find other targets of sEV in the colon next. In addition, M2 macrophages can be further classified into M2a, M2b, M2c, and M2d cells. Exosomes derived from M2b macrophages have been reported to alleviate IBD [60], so we also need to further explore whether it is M2b macrophages that engulf sEV and which roles other types of M2 macrophages play.

In many studies, MSCs and MSC-derived EVs had the same therapeutic effect on the same disease, which led many researchers to speculate that MSCs may play the therapeutic effect on diseases by secreting EVs [13, 61, 62]. The advantages of small EVs are their small size, more direct action, better therapeutic effect, and the ability to freely shuttle through the blood circulation without being trapped in capillaries [20]. Besides, previous researchers reported that EVs secreted by MSCs derived from the umbilical cord and bone marrow would not produce rejection in mice [17, 63]. In animal experiments, mice were treated with EVs by intraperitoneal injection. One of the reasons is that bubble generation during intravenous injections can lead to certain mortality in mice. The second reason is that drugs administrated intravenously are mainly in the lungs and liver, which is poorly absorbed by the colon [37].

IBD is a chronic and progressive intestinal inflammatory disease. TNF-*α* is a proinflammatory cytokine. In the disease process of IBD, TNF-*α* expression is substantially upregulated in the colon. Nowadays, many researchers tend to use TNF-*α* inhibitors to treat IBD [64–66]. Preconditioning MenSCs with TNF-*α* was used to mimic the inflammatory environment in the gut, forcing MenSCs to secrete substances to inhibit TNF-*α*. Previous investigators pretreated MenSCs with TNF-*α* and IFN-*γ*, and they verified that this pretreatment enhances the immunomodulatory capacity of MenSCs-derived EVs by sequencing and cellular experiments [67]. In addition, we also found that MenSCs-sEV^TNF-*α*^ decreased TNF-*α* expression both *in vivo* and *in vitro*. TNF-*α* is generally secreted by M1 macrophages, which helps macrophages to destroy microorganisms and phagocytose necrotic cells when the tissue is damaged by the invasion [2, 68, 69]. Macrophages, as a natural immune barrier, kill exogenous microorganisms and cause damage to adjacent tissues. Hyperinflammation caused by M1 macrophages is a crucial cause of IBD, and the transformation of M1 macrophages into M2 macrophages can alleviate inflammation in the colon [60, 70–72]. Research shows that exosomes are quickly cleared by the blood system whether they are injected intraperitoneally, intravenously, or subcutaneously [73]. Small EV clearance appeared to be regulated in part by the innate immune response and may be mediated by complementary conditioning [74]. Macrophages of the endothelial reticular system can rapidly phagocytose EVs, regardless of the mode of administration. Both adipose-derived and bone marrow-derived MSCs can promote the production of M2 macrophages by secreting small EVs, thereby alleviating inflammation [45, 75, 76]. IRFs are closely associated with the maturation, formation, phenotypic transformation, and phenotypic polarization of macrophages. In the IRF family, IRF1, IRF5, and IRF8 promote the production of proinflammatory macrophage M1, while IRF3 and IRF4 promote the production of regulatory macrophage M2. The regulatory effect of IRF2 changes with context [54]. In the resting state, the amount of IRF1 in macrophages is very low. Under the stimulation of the inflammatory environment, the content of IRF1 will increase, accompanied by an increase in the number of M1 macrophages [77]. As a result, IRF1 is an important target for MenSCs-sEV^TNF-*α*^ to influence the polarization of M2 macrophages.

## 5. Conclusion

Small EVs derived from TNF-*α*-pretreated MenSCs can relieve colonic inflammation. TNF-*α* can upregulate miR-24-3p in MenSCs-sEV^TNF-*α*^, and the microRNA can transform macrophages from M1 to M2 to relieve inflammation by binding to downstream IRF1 in DSS-induced IBD mice.

## Figures and Tables

**Table 1:** Diseases activity index scoring.

Weight loss	Score	Stool consistency	Score	Hematochezia	Score
None	0	Well-formed pellets	0	Negative fecal occult blood test	0
1-5%	1	Soft stool	1	Positive fecal occult blood test	1
6-10%	2	Loose stool	2	Blood visibly present in the stool	2
11-15%	3	Diarrhea	3	Blood visible and blood clotting on the anus	3
>15%	4			Rectal gross bleeding	4

**Table 2:** H&E staining scoring.

Inflammatory infiltration	Score	Tissue injury	Score	Crypt change	Score
None	0	None	0	None	0
Mild	1	Mucosal lesion	1	1/3 crypt near the basement membrane was damaged	1
Middle	2	Mucosa and submucosa lesion	2	2/3 crypt near the basement membrane was damaged	2
Severe	3	Whole mucosa lesion	3	Only epithelium intact	3
				All crypts and epithelium were destroyed	4

## Data Availability

All meaningful data of this study are presented in the results and supplements of this paper. More detailed data can be obtained by reasonably requesting the corresponding author.

## Abbreviations

EV: Extracellular vesicle
MenSCs: Menstrual blood-derived mesenchymal stem cells
MenSCs-sEV: MenSC-derived small extracellular vesicles
MenSCs-sEV^TNF-*α*^: TNF-*α*-pretreated MenSC-derived small extracellular vesicles
ZO: Zonula occludens
OE: Overexpression
IBD: Inflammatory bowel disease
UC: Ulcerative colitis
CD: Crohn's disease
TNF-*α*: Antitumor necrosis factor-*α*
MSCs: Mesenchymal stem cells
DSS: Dextran sulfate sodium
DAI: Disease activity index
DMSO: Dimethylsulfoxide
HLA-DR: Human leukocyte antigen-DR
TEM: Transmission electron microscopy
qPCR: Quantitative polymerase chain reaction
ELISA: Enzyme-linked immunosorbent assays
CCK8: Cell Counting Kit 8
TGF: Transforming growth factor
CTGF: Connective tissue growth factor
PDGF: Platelet-derived growth factor
FGF: Fibroblast growth factor
IGF: Insulin-like growth factor
FBS: Fetal bovine serum.

## Additional Points

*ARRIVE guidelines statement*. The authors have read the ARRIVE guidelines, and the manuscript was prepared and revised according to the ARRIVE guidelines.

## Supplementary Materials

Supplementary Materials Supplementary Figure S1: before mice were induced to acute IBD with DSS, 250 *μ*l clod-lipo or PBS-lipo were intraperitoneally injected to remove macrophages. (a–d) After the clearance of macrophages, MenSCs-sEV^TNF-*α*^ no longer played a therapeutic role in mouse colitis, which can be shown in the results of DAI, colon length, and colon pathology. (e) After removal of macrophages with clop-lipo, the proportion of macrophages in the bone marrow was detected by flow cytometry. Supplemental table 1: the RNA primers applied for qRT-PCR. Supplemental table 2: the differential expression results of sequencing were presented: there were 70 differential microRNAs, 38 of which were upregulated and 32 downregulated.
