# Supplementary material for: TNF-α Enhances the Therapeutic Effects of MenSC-Derived Small Extracellular Vesicles on Inflammatory Bowel Disease through Macrophage Polarization by miR-24-3p
Source: Stem Cells Int. 2023 Feb 28;2023:2988907. doi: 10.1155/2023/2988907 (PMC9991477; doi:10.1155/2023/2988907)
Supplement: Supplementary Materials — Supplementary Figure S1: before mice were induced to acute IBD with DSS, 250 μl clod-lipo or PBS-lipo were intraperitoneally injected to remove macrophages. (a–d) After the clearance of macrophages, MenSCs-sEVTNF-α no longer played a therapeutic role in mouse colitis, which can be shown in the results of DAI, colon length, and colon pathology. (e) After removal of macrophages with clop-lipo, the proportion of macrophages in the bone marrow was detected by flow cytometry. Supplemental table 1: the RNA primers applied for qRT-PCR. Supplemental table 2: the differential expression results of sequencing were presented: there were 70 differential microRNAs, 38 of which were upregulated and 32 downregulated. [file 2988907.f1.docx]

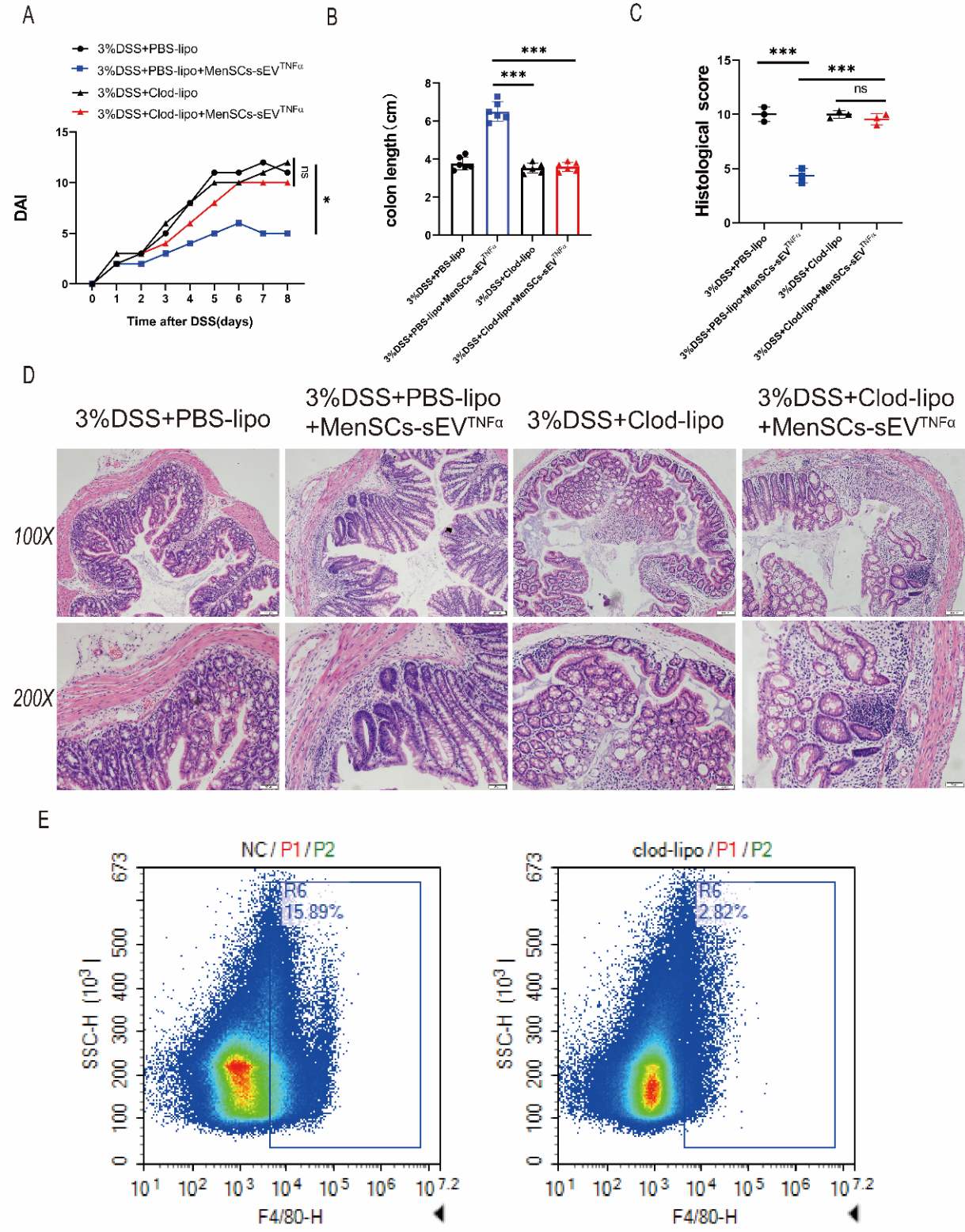


Supplementary Figure S1. Before mice were induced to acute IBD with DSS, 250ul Clophosome liposomes (clod-lipo) or PBS-liposomes (PBS-lipo) were intraperitoneally injected to remove macrophages. a, b, c, d. After the clearance of macrophages, MenSCs - sEV ^TNF-α^ no longer played a therapeutic role in mice colitis, which can be shown in the results of DAI, colon length, and colon pathology. e. After removal of macrophages with clop-lipo, the proportion of macrophages in bone marrow was detected by flow cytometry.

Supplemental table 1. The RNA primers applied for qRT-PCR.

| Gene name | Primer name | Species |
| --- | --- | --- |
| U6 | U6-F: CTCGCTTCGGCAGCACA  U6-R: AACGCTTCACGAATTTGCGT | human |
| miR-24-3P-RT | miR-24-3P-RT：GTCGTATCCAGTGCAGGGTCCGAGGT  ATTCGCACTGGATACGACCTGTTC | human |
| miR-24-3P | miR-24-3P-F: GCGTGGCTCAGTTCAGCAG  miR-24-3P-R: AGTGCAGGGTCCGAGGTATT | human |
| IRF1 | IRF1-F: ATGCCAATCACTCGAATGCG  IRF1-R: TTGTATCGGCCTGTGTGAATG | Mouse |
| iNOS | iNOS-F：GTTCTCAGCCCAACAATACAAGA  iNOS-R：GTGGACGGGTCGATGTCAC | Mouse |
| GAPDH | GAPDH-F: AGGTCGGTGTGAACGGATTTG  GAPDH-R: TGTAGACCATGTAGTTGAGGTCA | Mouse |
| TNFα | TNFα-F: CTGAACTTCGGGGTGATCGG  TNFα-R: GGCTTGTCACTCGAATTTTGAGA | Mouse |
| IL-1β | IL-1β-F: GAAATGCCACCTTTTGACAGTG  IL-1β-R: TGGATGCTCTCATCAGGACAG | Mouse |
| IFN-γ | IFN-γ-F: GCCACGGCACAGTCATTGA  IFN-γ-R: TGCTGATGGCCTGATTGTCTT | Mouse |
| IL-17 | IL-17-F: TCAGCGTGTCCAAACACTGAG  IL-17-R: CGCCAAGGGAGTTAAAGACTT | Mouse |
| IL-10 | IL-10-F: CTTACTGACTGGCATGAGGATCA  IL-10-R: GCAGCTCTAGGAGCATGTGG | Mouse |
| Arg1 | Arg1-F: CTCCAAGCCAAAGTCCTTAGAG  Arg1-R: GGAGCTGTCATTAGGGACATCA | Mouse |

Supplemental table 2. The differential expression results of sequencing were presented: there were 70 differential microRNAs, 38 of which were up-regulated and 32 down-regulated.

| miRNA | MENSCS1 | MENSCS2 | MENSCS3 | TNF_MEN_S1 | TNF_MEN_S2 | TNF_MEN_S3 | log2(Fold_change) | p-value | Website |
| --- | --- | --- | --- | --- | --- | --- | --- | --- | --- |
| hsa-miR-5100 | 0 | 0 | 0 | 9.308945 | 8.96403 | 1.709899 | 5.824372 | 6.17E-05 | http://www.mirbase.org/cgi-bin/mature.pl?mature_acc=MIMAT0022259 |
| hsa-miR-146a-5p | 10.96914 | 7.471302 | 4.464937 | 241.5188 | 252.6068 | 261.9679 | 4.936876 | 1.78E-37 | http://www.mirbase.org/cgi-bin/mature.pl?mature_acc=MIMAT0000449 |
| hsa-miR-9-3p | 0 | 0 | 0 | 0 | 2.702026 | 2.758349 | 4.013967 | 0.040976 | http://www.mirbase.org/cgi-bin/mature.pl?mature_acc=MIMAT0000442 |
| hsa-miR-4449 | 0 | 1.049066 | 0.044731 | 4.639689 | 4.458904 | 2.684421 | 3.142201 | 0.00672 | http://www.mirbase.org/cgi-bin/mature.pl?mature_acc=MIMAT0018968 |
| hsa-miR-576-5p | 0 | 2.090576 | 0.088057 | 8.396556 | 4.402822 | 3.581328 | 2.794282 | 0.002993 | http://www.mirbase.org/cgi-bin/mature.pl?mature_acc=MIMAT0003241 |
| hsa-miR-222-5p | 0 | 1.049066 | 0.044731 | 1.814291 | 4.503672 | 2.710866 | 2.77375 | 0.031711 | http://www.mirbase.org/cgi-bin/mature.pl?mature_acc=MIMAT0004569 |
| hsa-miR-181a-3p | 0 | 2.090576 | 0.088057 | 4.631159 | 6.300934 | 1.746489 | 2.435225 | 0.017152 | http://www.mirbase.org/cgi-bin/mature.pl?mature_acc=MIMAT0000270 |
| hsa-miR-31-5p | 0 | 3.133535 | 0.129963 | 2.714003 | 8.095497 | 5.450347 | 2.265135 | 0.015049 | http://www.mirbase.org/cgi-bin/mature.pl?mature_acc=MIMAT0000089 |
| hsa-miR-455-3p | 6.139893 | 21.24357 | 21.642 | 54.51158 | 67.5419 | 105.6604 | 2.237569 | 2.87E-05 | http://www.mirbase.org/cgi-bin/mature.pl?mature_acc=MIMAT0004784 |
| hsa-miR-34a-5p | 2.837491 | 0.022019 | 1.888535 | 5.506544 | 8.92891 | 8.194824 | 2.114741 | 0.004441 | http://www.mirbase.org/cgi-bin/mature.pl?mature_acc=MIMAT0000255 |
| hsa-miR-151b | 1.869473 | 3.191029 | 3.588611 | 7.32298 | 6.989802 | 22.95873 | 2.109605 | 0.00462 | http://www.mirbase.org/cgi-bin/mature.pl?mature_acc=MIMAT0010214 |
| hsa-miR-151a-5p | 1.156768 | 3.182577 | 3.506238 | 5.470042 | 6.116621 | 19.30884 | 2.001431 | 0.012032 | http://www.mirbase.org/cgi-bin/mature.pl?mature_acc=MIMAT0004697 |
| hsa-miR-409-5p | 1.163883 | 5.250933 | 0.276402 | 9.245284 | 5.223706 | 13.74056 | 1.932156 | 0.009313 | http://www.mirbase.org/cgi-bin/mature.pl?mature_acc=MIMAT0001638 |
| hsa-miR-708-5p | 2.842464 | 1.083757 | 0.197824 | 5.53461 | 1.652909 | 11.05807 | 1.860044 | 0.02713 | http://www.mirbase.org/cgi-bin/mature.pl?mature_acc=MIMAT0004926 |
| hsa-miR-30c-5p | 186.6866 | 602.694 | 170.412 | 1110.509 | 1048.657 | 1263.446 | 1.833522 | 0.000582 | http://www.mirbase.org/cgi-bin/mature.pl?mature_acc=MIMAT0000244 |
| hsa-miR-345-5p | 10.80198 | 11.68556 | 4.611035 | 28.65603 | 25.71181 | 41.0229 | 1.74271 | 7.46E-05 | http://www.mirbase.org/cgi-bin/mature.pl?mature_acc=MIMAT0000772 |
| hsa-miR-22-3p | 212.8008 | 280.0574 | 95.53446 | 561.6862 | 686.7015 | 715.8721 | 1.734561 | 6.19E-05 | http://www.mirbase.org/cgi-bin/mature.pl?mature_acc=MIMAT0000077 |
| hsa-let-7e-5p | 34.23377 | 25.61015 | 31.81724 | 51.68959 | 61.21314 | 168.6035 | 1.615248 | 0.002409 | http://www.mirbase.org/cgi-bin/mature.pl?mature_acc=MIMAT0000066 |
| hsa-miR-134-5p | 77.11869 | 64.99711 | 56.80052 | 217.4916 | 200.1133 | 198.2671 | 1.613373 | 5.03E-09 | http://www.mirbase.org/cgi-bin/mature.pl?mature_acc=MIMAT0000447 |
| hsa-miR-574-3p | 107.2481 | 261.8544 | 89.60152 | 508.0885 | 475.8759 | 420.1768 | 1.611485 | 0.000483 | http://www.mirbase.org/cgi-bin/mature.pl?mature_acc=MIMAT0003239 |
| hsa-miR-93-5p | 24.56592 | 18.09949 | 5.099812 | 39.74306 | 54.3173 | 47.29181 | 1.507839 | 0.001679 | http://www.mirbase.org/cgi-bin/mature.pl?mature_acc=MIMAT0000093 |
| hsa-miR-30b-5p | 2.992491 | 11.62353 | 5.879474 | 13.81149 | 13.22906 | 31.10538 | 1.507297 | 0.016723 | http://www.mirbase.org/cgi-bin/mature.pl?mature_acc=MIMAT0000420 |
| hsa-miR-199a-5p | 504.1597 | 722.2125 | 458.1114 | 1452.9 | 1782.59 | 1538.989 | 1.501841 | 4.67E-07 | http://www.mirbase.org/cgi-bin/mature.pl?mature_acc=MIMAT0000231 |
| hsa-miR-181a-5p | 545.7378 | 788.2256 | 353.813 | 1345.533 | 1524.564 | 1806.488 | 1.468649 | 7.30E-05 | http://www.mirbase.org/cgi-bin/mature.pl?mature_acc=MIMAT0000256 |
| hsa-miR-331-3p | 7.282208 | 13.74136 | 0.694568 | 15.67177 | 22.25804 | 23.68711 | 1.408773 | 0.0216 | http://www.mirbase.org/cgi-bin/mature.pl?mature_acc=MIMAT0000760 |
| hsa-miR-24-2-5p | 13.92501 | 20.18611 | 11.83889 | 34.18833 | 41.80375 | 47.34424 | 1.404105 | 0.000271 | http://www.mirbase.org/cgi-bin/mature.pl?mature_acc=MIMAT0004497 |
| hsa-miR-181b-5p | 342.6001 | 391.9679 | 196.3408 | 709.7675 | 742.6806 | 933.2971 | 1.353698 | 0.000108 | http://www.mirbase.org/cgi-bin/mature.pl?mature_acc=MIMAT0000257 |
| hsa-miR-214-3p | 63.44225 | 84.09643 | 53.24769 | 162.8776 | 152.0705 | 163.7274 | 1.242664 | 2.52E-05 | http://www.mirbase.org/cgi-bin/mature.pl?mature_acc=MIMAT0000271 |
| hsa-miR-1260b | 18.55272 | 9.626289 | 6.533715 | 17.4857 | 22.14835 | 44.76522 | 1.236936 | 0.019116 | http://www.mirbase.org/cgi-bin/mature.pl?mature_acc=MIMAT0015041 |
| hsa-miR-23a-3p | 429.5456 | 1102.423 | 507.1007 | 1575.084 | 1600.185 | 1593.579 | 1.225462 | 0.003233 | http://www.mirbase.org/cgi-bin/mature.pl?mature_acc=MIMAT0000078 |
| hsa-miR-30e-5p | 22.98809 | 23.38616 | 6.957543 | 33.2554 | 45.39626 | 45.50638 | 1.168886 | 0.008606 | http://www.mirbase.org/cgi-bin/mature.pl?mature_acc=MIMAT0000692 |
| hsa-miR-342-3p | 9.522254 | 27.61255 | 22.19158 | 32.32422 | 48.99298 | 42.75434 | 1.089159 | 0.018874 | http://www.mirbase.org/cgi-bin/mature.pl?mature_acc=MIMAT0000753 |
| hsa-miR-24-3p | 2377.186 | 3080.863 | 1736.712 | 4976.986 | 5793.305 | 4535.115 | 1.088563 | 0.000555 | http://www.mirbase.org/cgi-bin/mature.pl?mature_acc=MIMAT0000080 |
| hsa-miR-199b-5p | 40.88155 | 110.657 | 71.42351 | 107.2455 | 155.6449 | 210.2457 | 1.087405 | 0.018587 | http://www.mirbase.org/cgi-bin/mature.pl?mature_acc=MIMAT0000263 |
| hsa-miR-193b-3p | 29.41926 | 34.05547 | 23.13865 | 66.65286 | 60.47087 | 56.35784 | 1.061975 | 0.001042 | http://www.mirbase.org/cgi-bin/mature.pl?mature_acc=MIMAT0002819 |
| hsa-miR-197-3p | 38.73273 | 51.06907 | 29.0037 | 71.20738 | 83.60347 | 95.55501 | 1.057862 | 0.001702 | http://www.mirbase.org/cgi-bin/mature.pl?mature_acc=MIMAT0000227 |
| hsa-miR-379-5p | 47.43913 | 67.01726 | 29.36924 | 80.4497 | 81.74486 | 133.8463 | 1.028033 | 0.012958 | http://www.mirbase.org/cgi-bin/mature.pl?mature_acc=MIMAT0000733 |
| hsa-miR-181d-5p | 10.1738 | 9.573088 | 4.511696 | 11.95945 | 16.88426 | 22.8255 | 1.021229 | 0.02841 | http://www.mirbase.org/cgi-bin/mature.pl?mature_acc=MIMAT0002821 |
| hsa-miR-148a-3p | 999.0514 | 882.1773 | 1686.981 | 628.3858 | 578.1452 | 574.8341 | -1.00079 | 0.002921 | http://www.mirbase.org/cgi-bin/mature.pl?mature_acc=MIMAT0000243 |
| hsa-miR-335-5p | 124.9875 | 188.4367 | 78.30034 | 52.6872 | 90.01221 | 52.67956 | -1.00588 | 0.021933 | http://www.mirbase.org/cgi-bin/mature.pl?mature_acc=MIMAT0000765 |
| hsa-miR-101-3p | 29.65737 | 23.45154 | 19.36966 | 12.02804 | 10.63638 | 12.74555 | -1.0544 | 0.011494 | http://www.mirbase.org/cgi-bin/mature.pl?mature_acc=MIMAT0000099 |
| hsa-miR-23b-5p | 15.38972 | 8.599031 | 21.47842 | 5.522451 | 4.369474 | 10.09473 | -1.14728 | 0.038762 | http://www.mirbase.org/cgi-bin/mature.pl?mature_acc=MIMAT0004587 |
| hsa-miR-576-3p | 12.09029 | 8.563963 | 12.87392 | 3.668473 | 7.198549 | 3.595969 | -1.1801 | 0.032836 | http://www.mirbase.org/cgi-bin/mature.pl?mature_acc=MIMAT0004796 |
| hsa-miR-432-5p | 158.4709 | 152.3646 | 174.0795 | 67.54413 | 67.5825 | 71.86489 | -1.223 | 3.19E-06 | http://www.mirbase.org/cgi-bin/mature.pl?mature_acc=MIMAT0002814 |
| hsa-miR-1246 | 210.0238 | 154.5498 | 257.737 | 92.57073 | 86.28475 | 72.70925 | -1.30102 | 4.44E-05 | http://www.mirbase.org/cgi-bin/mature.pl?mature_acc=MIMAT0005898 |
| hsa-miR-485-5p | 85.90065 | 32.09303 | 94.40981 | 26.81634 | 24.84791 | 33.70752 | -1.30809 | 0.006017 | http://www.mirbase.org/cgi-bin/mature.pl?mature_acc=MIMAT0002175 |
| hsa-miR-423-5p | 1590.282 | 866.2169 | 1431.581 | 616.4301 | 442.9111 | 401.0464 | -1.41251 | 6.86E-05 | http://www.mirbase.org/cgi-bin/mature.pl?mature_acc=MIMAT0004748 |
| hsa-miR-619-5p | 23.95526 | 19.23766 | 36.38419 | 11.13225 | 7.95759 | 9.081239 | -1.45824 | 0.001283 | http://www.mirbase.org/cgi-bin/mature.pl?mature_acc=MIMAT0026622 |
| hsa-miR-505-5p | 9.531667 | 5.368592 | 7.576573 | 3.736144 | 0 | 3.677891 | -1.59561 | 0.027064 | http://www.mirbase.org/cgi-bin/mature.pl?mature_acc=MIMAT0004776 |
| hsa-miR-3960 | 16.44705 | 4.351326 | 12.90916 | 5.602232 | 1.703309 | 3.630425 | -1.59962 | 0.022825 | http://www.mirbase.org/cgi-bin/mature.pl?mature_acc=MIMAT0019337 |
| hsa-miR-574-5p | 15.56296 | 22.41746 | 46.59138 | 7.366339 | 12.54725 | 7.228597 | -1.6028 | 0.003976 | http://www.mirbase.org/cgi-bin/mature.pl?mature_acc=MIMAT0004795 |
| hsa-miR-224-5p | 526.1155 | 661.5701 | 745.3813 | 238.8063 | 189.4071 | 197.3498 | -1.62505 | 8.09E-09 | http://www.mirbase.org/cgi-bin/mature.pl?mature_acc=MIMAT0000281 |
| hsa-miR-3182 | 57.6804 | 40.53827 | 54.20346 | 20.42607 | 7.854686 | 18.23219 | -1.70665 | 4.88E-05 | http://www.mirbase.org/cgi-bin/mature.pl?mature_acc=MIMAT0015062 |
| hsa-miR-365a-5p | 14.17044 | 10.67949 | 11.45109 | 0.863498 | 4.472909 | 5.515264 | -1.72023 | 0.004362 | http://www.mirbase.org/cgi-bin/mature.pl?mature_acc=MIMAT0009199 |
| hsa-miR-369-3p | 16.12959 | 4.327406 | 2.814806 | 2.790769 | 2.685168 | 0.864338 | -1.85781 | 0.034173 | http://www.mirbase.org/cgi-bin/mature.pl?mature_acc=MIMAT0000721 |
| hsa-miR-4448 | 54.17311 | 29.93754 | 77.1869 | 16.70577 | 15.15505 | 8.089138 | -1.99538 | 6.54E-05 | http://www.mirbase.org/cgi-bin/mature.pl?mature_acc=MIMAT0018967 |
| hsa-miR-9901 | 20.40467 | 6.488077 | 18.30588 | 1.821113 | 5.457654 | 0.850704 | -2.41966 | 0.000627 | http://www.mirbase.org/cgi-bin/mature.pl?mature_acc=MIMAT0039321 |
| hsa-miR-27a-5p | 87.61781 | 126.8537 | 307.8985 | 32.40052 | 36.54037 | 22.65222 | -2.50525 | 5.76E-06 | http://www.mirbase.org/cgi-bin/mature.pl?mature_acc=MIMAT0004501 |
| hsa-miR-10396b-5p | 1.864937 | 2.150903 | 5.008878 | 0.942378 | 0 | 0 | -2.78519 | 0.032499 | http://www.mirbase.org/cgi-bin/mature.pl?mature_acc=MIMAT0041635 |
| hsa-miR-490-5p | 2.593552 | 4.236919 | 2.17267 | 0 | 0 | 0.929659 | -2.90002 | 0.031233 | http://www.mirbase.org/cgi-bin/mature.pl?mature_acc=MIMAT0004764 |
| hsa-miR-19a-3p | 5.314891 | 5.333709 | 5.64721 | 0 | 0 | 1.870566 | -2.92906 | 0.003581 | http://www.mirbase.org/cgi-bin/mature.pl?mature_acc=MIMAT0000073 |
| hsa-miR-10396a-5p | 3.7932 | 4.293665 | 10.15776 | 1.894022 | 0 | 0 | -3.02122 | 0.002465 | http://www.mirbase.org/cgi-bin/mature.pl?mature_acc=MIMAT0041623 |
| hsa-miR-1268a | 5.54512 | 4.257646 | 0.412971 | 0 | 0.913294 | 0 | -3.21298 | 0.023707 | http://www.mirbase.org/cgi-bin/mature.pl?mature_acc=MIMAT0005922 |
| hsa-miR-372-3p | 21.36847 | 0.071359 | 0.73367 | 0.942378 | 0 | 0 | -4.0873 | 0.030062 | http://www.mirbase.org/cgi-bin/mature.pl?mature_acc=MIMAT0000724 |
| hsa-miR-3129-5p | 3.568806 | 0.025498 | 1.96008 | 0 | 0 | 0 | -4.1092 | 0.042091 | http://www.mirbase.org/cgi-bin/mature.pl?mature_acc=MIMAT0014992 |
| hsa-miR-10394-3p | 1.964948 | 0.026749 | 4.775423 | 0 | 0 | 0 | -4.21737 | 0.025202 | http://www.mirbase.org/cgi-bin/mature.pl?mature_acc=MIMAT0041620 |
| hsa-miR-122-5p | 386.0043 | 74.75699 | 639.6846 | 11.02923 | 21.43959 | 17.28572 | -4.45843 | 1.75E-08 | http://www.mirbase.org/cgi-bin/mature.pl?mature_acc=MIMAT0000421 |
| hsa-miR-4435 | 5.661878 | 0.038128 | 3.705791 | 0 | 0 | 0 | -4.79381 | 0.00931 | http://www.mirbase.org/cgi-bin/mature.pl?mature_acc=MIMAT0018951 |
| hsa-miR-4324 | 4.878048 | 2.159578 | 2.210784 | 0 | 0 | 0 | -4.81874 | 0.006479 | http://www.mirbase.org/cgi-bin/mature.pl?mature_acc=MIMAT0016876 |
| hsa-miR-6770-5p | 1.809282 | 0.038455 | 9.320105 | 0 | 0 | 0 | -5.03517 | 0.01227 | http://www.mirbase.org/cgi-bin/mature.pl?mature_acc=MIMAT0027440 |
